# Investigating experiences of frequent online food delivery service use: a qualitative study in UK adults

**DOI:** 10.1186/s12889-022-13721-9

**Published:** 2022-07-16

**Authors:** Matthew Keeble, Jean Adams, Thomas Burgoine

**Affiliations:** MRC Epidemiology Unit, University of Cambridge School of Clinical Medicine, Box 285 Institute of Metabolic Science, Cambridge Biomedical Campus, Cambridge, CB2 0QQ UK

**Keywords:** Diet, Fast foods, Meal delivery, Online food delivery services, Qualitative methods, Thematic analysis

## Abstract

**Background:**

Food prepared out-of-home is typically energy-dense and nutrient-poor. This food can be purchased from multiple types of retailer, including restaurants and takeaway food outlets. Using online food delivery services to purchase food prepared out-of-home is increasing in popularity. This may lead to more frequent unhealthy food consumption, which is positively associated with poor diet and living with obesity. Understanding possible reasons for using online food delivery services might contribute to the development of future public health interventions, if deemed necessary. This knowledge would be best obtained by engaging with individuals who use online food delivery services as part of established routines. Therefore, we aimed to investigate customer experiences of using online food delivery services to understand their reasons for using them, including any advantages and drawbacks.

**Methods and results:**

In 2020, we conducted telephone interviews with 22 adults living in the UK who had used online food delivery services on at least a monthly basis over the previous year. Through codebook thematic analysis, we generated five themes: ‘The importance of takeaway food’, ‘Less effort for more convenience’, ‘Saving money and reallocating time’, ‘Online food delivery service normalisation’ and ‘Maintained home food practices’. Two concepts were overarching throughout: ‘Place. Time. Situation.’ and ‘Perceived advantages outweigh recognised drawbacks’.

After considering each of the accessible food purchasing options within the context of their location and the time of day, participants typically selected online food delivery services. Participants reported that they did not use online food delivery services to purchase healthy food. Participants considered online food delivery service use to be a normal practice that involves little effort due to optimised purchasing processes. As a result, these services were seen to offer convenient access to food aligned with sociocultural expectations. Participants reported that this convenience was often an advantage but could be a drawback. Although participants were price-sensitive, they were willing to pay delivery fees for the opportunity to complete tasks whilst waiting for delivery. Furthermore, participants valued price-promotions and concluded that receiving them justified their online food delivery service use. Despite takeaway food consumption, participants considered home cooking to be irreplaceable.

**Conclusions:**

Future public health interventions might seek to increase the healthiness of food available online whilst maintaining sociocultural values. Extending restrictions adopted in other food environments to online food delivery services could also be explored.

**Supplementary Information:**

The online version contains supplementary material available at 10.1186/s12889-022-13721-9.

## Background

Purchasing food that is prepared out-of-home and served ready-to-consume is prevalent across the world [1]. The neighbourhood food environment includes all physically accessible food outlets where individuals can purchase and consume foods, including food prepared out-of-home (often referred to as ‘takeaway food’) [2]. An increased number of outlets selling this food in the neighbourhood food environment may have contributed to normalising its consumption [3]. Purchasing formats represent ways to buy takeaway food. Although the opportunity to purchase this food was once limited to visiting food outlets in person or placing orders directly with food outlets by phone, additional purchasing formats such as online food delivery services now exist [4]. Unlike physically accessing outlets in the neighbourhood food environment or contacting outlets by telephone before collection or delivery, online food delivery services exist within a digital food environment. On a single online platform, customers receive aggregated information about food outlets that will deliver to them based on their location. Customers then select a food outlet, and place and pay for their order. Orders are forwarded to food outlets where meals are prepared before being delivered to customers [5]. Online food delivery services have been available in the UK since around 2006. However, widespread internet and smartphone access has increased their use [6], with global online food delivery service revenue estimated at £2.9 billion in 2021 [7]. The COVID-19 pandemic may have accelerated and perpetuated market development [8].

Food sold by takeaway food outlets, and therefore available online, is typically nutrient-poor and served in portion sizes that exceed public health recommendations for energy content [9, 10]. More frequent takeaway food consumption has been associated with poorer diet quality and elevated bodyweight over time [11]. Although it is currently unclear, using online food delivery services might lead to more frequent and higher overall takeaway food consumption. In turn, this could lead to increased risk of elevated bodyweight and associated comorbidities. Since an estimated 67% of men and 60% of women in the UK were already considered overweight or obese in 2019 [12], the possibility that using online food delivery services increases overall takeaway food consumption is a major public health concern, as recognised by the World Health Organization [4, 13, 14].

With respect to the neighbourhood food environment, food outlet accessibility (number) and proximity (distance to nearest), food availability (presence of variety), and attitudinal dimensions (acceptability) contribute to takeaway food purchasing practices [15]. Each of these domains apply to takeaway food access through online food delivery services. In 2019, the number of food outlets accessible through the leading online food delivery service in the UK (*Just Eat*) was 50% greater in the most deprived areas compared with the least deprived areas [16]. Furthermore, adults living in the UK with the highest number of food outlets accessible online had greater odds of any online delivery service use in the previous week compared to those with the lowest number [17]. To our knowledge, however, attitudinal dimensions of online food delivery service use have not been investigated in the public health literature. Given the complexity of takeaway food purchasing practices, there are likely to be unique and specific reasons for using online food delivery services. Understanding these reasons from the perspective of customers could contribute to more informed public health decision-making and intervention, which is important since public health interventions that include online food delivery services may be increasingly necessary as their growth in popularity continues worldwide [13, 18].

In our study, we investigated experiences of using online food delivery services from the perspective of adults living in the UK who use them frequently. We aimed to understand their reasons for using these services, the possible advantages and drawbacks of doing so, and how they coexist with other food-related practices.

## Methods

Between June and August 2020, we used semi-structured telephone interviews to study experiences of using online food delivery services from the perspective of adults living in the UK. We used the Consolidated Criteria for Reporting Qualitative Research (COREQ) checklist to guide the development and reporting of our study [19].

The University of Cambridge School of the Humanities and Social Sciences Research Ethics Committee provided ethical approval (Reference: 19/220).

### Methodological orientation

We used a qualitative description methodological orientation to investigate our study aims. Qualitative description has been framed as less interpretative than other approaches [20]. However, it is theoretically and epistemologically flexible and can facilitate a rich description of perspectives [21], which matched our study aims.

### Participants and recruitment

We used convenience sampling to recruit adults that used online food delivery services frequently. For the purpose of our study, we defined frequent customers as those who had used online food delivery services on at least a monthly basis over the previous year. We believed this level of use would make participants well-positioned to provide their experiences of using this purchasing format within established takeaway food purchasing practices. We also based participant recruitment on reported sociodemographic characteristics of online food delivery service customers [22, 23]. As data collection progressed, we additionally considered level of education so that our sample included frequent customers who were less highly educated (see Table 1).

**Table 1:** Participant recruitment inclusion criteria

Able to communicate in English
Aged between 18 and 50 years
Living in the UK
Non-university educated*

^*^ We introduced this criteria after 12 participants had been recruited so that our sample included participants with different levels of education

We used two social media platforms (Twitter and Reddit) to recruit participants. Participant recruitment through social media platforms can be fast and efficient [24–26]. If targeted advertising is not used (as in our study), participant recruitment in this way is also typically free. In our study, participant recruitment through social media was particularly appropriate, given that our aims were related to understanding experiences of using a digital purchasing format. Twitter users can publish and re-publish information, images, videos, and links to external sites. Reddit users can publish information, images and videos, and discuss topics within focused forums known as ‘Subreddits’. For Twitter, the primary researcher (MK) published recruitment materials using his personal account and relied on existing connections to re-publish them. For Reddit, MK created an alias account (he did not have a personal account at the time of our fieldwork) and published recruitment materials in Subreddits for cities in the UK with large populations according to the 2011 UK census, those related to online food delivery services, and those that discuss topics relevant to the UK [27]. See Additional file 1 (Box A1) for a complete list of Subreddits.

Recruitment materials asked interested individuals to contact MK by email. When contacted, MK responded by email with screening questions that asked about self-reported frequency of online food delivery service use over the past year, age, and level of education. When eligibility was confirmed, MK provided information about the study by email. This information included the study aims, details about researchers involved, the offer of a £20.00 electronic high street shopping voucher, and a formal invitation to participate. After five business days with no response to the invitation, MK sent a further email. After another five business days, we classified individuals that did not respond as ‘non-respondents’.

### Data collection

#### Before data collection

Before starting data collection, we planned to complete a maximum of 25 interviews. We did not target data saturation. Food purchasing and consumption are highly individual and influenced by previous experiences, cultural backgrounds, and preferences [28]. Therefore, we felt that it would be difficult to conclude data saturation was achieved based on the traditional conceptualisation of no new information being reported by participants [29, 30]. Instead, we prioritised conceptual depth and information strength. This approach was aligned with the qualitative description methodological orientation of our study [30].

We wanted to investigate experiences of using online food delivery services from before the COVID-19 pandemic, when there were no restrictions on accessing multiple purchasing formats or consuming food on the premises. Therefore, we pre-specified that we would stop data collection if it became difficult for participants to refer to the time before March 2020, which is when pandemic related travel and food outlet access restrictions were first introduced in the UK. MK piloted an initial protocol with an eligible individual to confirm this would be possible, and made amendments based on their feedback.

Before starting data collection, MK reflected on his position as a population health researcher, and his previous training and experience in qualitative research [31]. MK also reflected on his own takeaway food consumption and previous use of online food delivery services. As of June 2020, MK consumed takeaway food infrequently and had previously placed one order with an online food delivery service. Although he was not a frequent customer according to our classification, MK was familiar with online food delivery services operating in the UK. MK concluded that despite having a broad understanding about why online food delivery services might be used, he could not use his own experiences to provide detailed reasons for favouring this purchasing format over alternative options.

#### Throughout data collection

MK completed one-off semi-structured telephone interviews with participants at a convenient time selected by them. At the start of the interview process, MK confirmed the rationale for the study, gave participants the opportunity to ask clarifying questions and asked them to provide verbal consent. MK used a topic guide that was developed based on a priori knowledge, pilot interview feedback and previous research related to takeaway food and online food delivery services [22, 32, 33]. MK amended the topic guide as data collection progressed so that points not initially considered could be discussed in future interviews. Interview questions focused on reasons for using online food delivery services, the perceived advantages and drawbacks of using these services, and how using them coexisted with other purchasing formats and food-related practices (see Box A2 in Additional file 1 for the final topic guide).

Although MK completed interviews during the COVID-19 pandemic, he did not ask questions related to this period of time, and prompted participants to think about the time before March 2020 so that pre-pandemic experiences could be discussed. MK digitally recorded interview audio and made field notes to track points for discussion within the interview.

#### After data collection

MK immediately reflected on topics discussed, data collection progress, possible links with existing theory, and the ability of participants to think about the time before the COVID-19 pandemic. We used these post-interview reflections to help inform our decision to stop data collection.

### Data analysis

A professional company transcribed interview audio verbatim. Whilst listening to the corresponding audio, MK quality assured each transcript and anonymised it. Participants did not review their transcripts.

We used codebook thematic analysis. When using this analytic approach, researchers develop a codebook based on the final topic guide used during data collection and data familiarity that is achieved by reviewing collected data [34, 35]. Codebook thematic analysis is aligned with qualitative description methodological orientations as it allows researchers to remain ‘close to the data’ and facilitates an understanding of a topic through the ‘spoken word’ of participants [36]. In practice, MK developed an initial codebook. MK, JA, and TB then reviewed three transcripts (a 10% sample). This number was manageable and allowed us to discuss a sample of collected data [37]. After discussion, MK refined the initial codebook to collapse codes that overlapped and to add new codes, which formed the final codebook. MK coded each transcript with the final codebook and reviewed reflections written after each interview. MK then studied the coded data to generate themes that were discussed and finalised with JA and TB. In the context of our study, themes summarise experiences of using online food delivery services from the perspective of participants. After discussion, we also identified that across the themes we generated, there were overarching concepts. For our study, concepts should be seen to offer an overall and consistent structure that capture the common and overlapping elements of each of the generated themes.

MK used NVivo (version 12) to manage the data and facilitate interpretation.

## Results

### Participant and data overview

MK conducted interviews with 22 frequent online food delivery service customers between June and August 2020. Interviews lasted between 35 and 61 min. There were 12 male participants, 13 participants were aged between 20 and 29 years, and 15 had completed higher education. Since initial adoption, participants had typically used online food delivery services at least fortnightly but as often as daily, and during interviews they consistently referred to using the three most well-established online food delivery services operating in the UK (*Just Eat, Deliveroo, and Uber Eats*) (see Table 2).

**Table 2:** Characteristics and use of online food delivery services for frequent customers interviewed between June and August 2020 (*n*=22)

**Participant**	**Gender**	**Age** (years)	**Highest education** ^a^	**Service(s) used**	**Typical usage frequency** ^b^	**Time since adoption** (years)
1	Female	20–29	Higher	Deliveroo & Just Eat	Monthly	≤ 5
2	Male	20–29	Higher	Deliveroo, Just Eat & Uber Eats	Fortnightly	> 5
3	Female	20–29	Higher	Deliveroo	Weekly	Unknown
4	Female	30–39	Higher	Deliveroo & Just Eat	Weekly	≤ 5
5	Female	30–39	Higher	Deliveroo & Just Eat	Weekly	≤ 5
6	Female	20–29	Higher	Deliveroo & Just Eat	Monthly	Unknown
7	Female	20–29	Higher	Deliveroo	Weekly	≤ 5
8	Female	40–49	Higher	Just Eat	Monthly	≤ 5
9	Male	30–39	Compulsory	Deliveroo, Just Eat & Uber Eats	Weekly	> 5
10	Female	20–29	Further	Deliveroo	Monthly	≤ 5
11	Male	30–39	Higher	Just Eat	Monthly	> 5
12	Female	20–29	Higher	Deliveroo, Just Eat & Uber Eats	Fortnightly	> 5
13	Male	20–29	Compulsory	Deliveroo, Just Eat & Uber Eats	Weekly	≤ 5
14	Female	20–29	Higher	Just Eat	Monthly	> 5
15	Male	40–49	Further	Just Eat	Monthly	≤ 5
16	Male	20–29	Higher	Deliveroo & Uber Eats	Fortnightly	≤ 5
17	Male	30–39	Higher	Deliveroo	Monthly	> 5
18	Male	20–29	Higher	Just Eat	Fortnightly	> 5
19	Male	30–39	Further	Deliveroo	Weekly	≤ 5
20	Male	20–29	Compulsory	Deliveroo & Just Eat	Fortnightly	≤ 5
21	Male	20–29	Compulsory	Deliveroo & Uber Eats	Daily	≤ 5
22	Male	30–39	Higher	Deliveroo & Uber Eats	Weekly	> 5

^a^ Highest level achieved or underway. ‘Compulsory’ = High school, ‘Further’ = Education after high school, not including a university degree, ‘Higher’ = University degree

^b^ Since initial adoption

During the 19^th^ interview, conducted in August 2020, it was difficult for the participant to think about the time before the onset of the COVID-19 pandemic in March 2020. MK completed three further interviews and then concluded that this difficulty was consistent so stopped data collection. We included data from all interviews in analyses. In addition to those who took part, three interviews were scheduled but cancelled by individuals without providing a reason, and there were nine non-respondents.

### Summary and structure

We generated two concepts that were overarching throughout our data: ‘Place. Time. Situation.’ and ‘Perceived advantages outweigh recognised drawbacks’. Within these overarching concepts, we generated five themes: ‘The importance of takeaway food’, ‘Less effort for more convenience’, ‘Saving money and reallocating time’, ‘Online food delivery service normalisation’ and ‘Maintained home food practices’.

In the following sections, we present the findings for each of the overarching concepts, followed by each of the themes. Whilst we discuss each concept and theme in turn, all of their elements were present throughout the data and should be thought of as dynamic, overlapping, and non-hierarchical. For example, participants consistently reflected on features of online food delivery services within the context of their location at a specific time. The conclusion of this process dictated whether a feature was viewed as an advantage or a drawback, and in some cases whether an online food delivery service would be used. We provide examples of this comparison process at the end of our Results (Table 3).

**Table 3:** Examples of how participants compared the advantages and drawbacks of online food delivery service features

Feature	Perceived advantage	Perceived drawback
Food outlet information and menus can be viewed, and orders placed, on one platform	Orders can be placed with little effort	It is *too easy,* and it takes no effort to purchase takeaway food
A greater number of food outlets are accessible compared with other purchasing formats	Food outlets, cuisines, and price points, including those not normally available, can be selected	Selecting a food outlet is difficult because there is *too much* choice
Unique promotional offers can be used	Money can be saved, additional food items can be received, and meals can be delivered for free	It is *too appealing* to place orders when promotional offers are available
‘Takeaway food’ can be purchased	The available food meets expectations	The available food is mostly unhealthy
Meals are delivered	Takeaway food can be received without leaving home	Having takeaway food delivered when it is nearby might be lazy
Delivery typically involves an additional fee	Paying a delivery fee is worth it to carry out other tasks whilst waiting	Delivery fees can be expensive

### Overarching concepts

#### Place. Time. Situation.

Participants described how their location and the time of day impacted their ability to access different types of food, including both ‘takeaway’ food and other types of food. When choosing one type of food over another, participants had a multi-factorial thought process that considered their food at home, immediate finances available for food, and the food already eaten that day.

Although data collection focused on takeaway food, participants were clear that this type of food was not always appropriate. As participant 10 (Female: 20–29 years) stated; “*I don’t always just go and get a takeaway; sometimes I’ll walk to the shop, get some food, and make something*”. This view was shared by participant 11 (Male 30–39 years); “*some days I’ll decide that it’s too expensive and I’ll either get something else direct from the restaurant or go to the supermarket and then make food*”.

Nonetheless, participants indicated that purchasing takeaway food was preferable in many situations. For example, when acting spontaneously, when meals had not been planned or if other types of food could not satisfy needs, then takeaway food was appropriate.

“*I think you’re more likely to get delivery and order online when it’s unplanned and you need a pick-me-up, or you need something quick, or you don’t have something and you’re really hungry*.” Participant 15 (Male: 40-49 years)

When participants decided to purchase takeaway food, they recognised that their location and the time of day dictated the purchasing formats they could access and potentially use. Access to multiple purchasing formats created a second decision making process. Participants considered the cuisines they wanted, delivery times estimated by online food delivery services versus the time it would take to travel to a food outlet, the weather, their willingness to leave home, and previous experience with accessible food outlets. Alongside these influential factors, choosing one purchasing format over another was often based on what was most convenient.

“*If I’m out and about, on the way home and I’m passing via an outlet, then I’ll pick it up. If I’m at home and just kind of, don’t want to leave the house, then I’ll order via an app or online, just because it’s just convenient*.” Participant 2 (Male: 20-29 years)

Despite having apparently decided how they would purchase takeaway food, participants stated that they could change their mind. In the case of online food delivery services, if estimated delivery times failed to meet expectations, this purchasing format would no longer be appropriate and another purchasing format or type of food would be selected. The need for food practices to align with other routines and schedules, and therefore meet expectations, was particularly clear when participant 8 (Female: 40–49 years) described that they used online food delivery services when they could “*relax on a Friday night with the whole evening free*”. However, if they do not have time to select a food outlet, place their order, and then wait for delivery they “*normally just have some spaghetti because that takes 10 min*”.

Participants also referred to online food delivery service marketing in their day-to-day environments, including branded food outlet signs and equipment used by delivery couriers. Participants stated that these things did not always trigger immediate use of online food delivery services, however, their omnipresence reminded them that these services were available.

“*I don’t know if I ever go onto Just Eat after seeing it advertised, I don’t think that’s ever directly led me to do it. But it certainly keeps it in your mind, it’s certainly at the forefront of your mind whenever you think of takeaway*.” Participant 11 (Male: 30-39 years)

#### Perceived advantages outweigh recognised drawbacks

Throughout the data, participants recognised that a single online food delivery service feature could be an advantage or a drawback based on their location and the time of day. This was clearest when participant 2 (Male: 20–29 years) discussed the number of food outlets accessible online compared with those available through other purchasing formats. There was value in having access to “*20, 30, 40 food outlets*” through online food delivery services as it meant more options, otherwise “*you’re more limited just by the virtue of where you are or what shops you’re passing*”. However, access to a greater number of food outlets was a drawback when it meant that making a selection was difficult. The constant comparison of advantages and drawbacks prompted MK to ask participants why they kept using online food delivery services. There was a consensus that features of these services were unique, mostly advantageous, and outweighed the instances where they were seen as drawbacks. Since participants continued to use online food delivery services to access unique features, this practice appears to be self-reinforcing, even if this means accepting that the same feature can sometimes be a drawback.

Participants favoured online food delivery services in many situations. Nevertheless, they acknowledged that if the overall balance between advantages and drawbacks changed then they would purchase takeaway food in other ways. This solution emphasises that takeaway food can often be accessed in multiple ways dependent on place and time. As it stands, participants anticipated that they would continue to use online food delivery services indefinitely.

“*I can’t see any reason why I would* [stop using online food delivery services]*, unless something went wrong with Just Eat, you know, the service had a massive problem, but at the moment I can’t see any reason why I would.*” Participant 16 (Male: 20-29 years)

### Analytic themes

We now present each of the five themes generated from our analyses. As described, elements of each theme overlapped within the two overarching concepts presented above.

#### The importance of takeaway food

Participants emphasised that, ultimately, it was “*the food*” that they valued, and that directed them towards using online food delivery services.

“*It’s the food really, that leads me to use* [online food delivery service] *apps*.” Participant 10 (Female: 20-29 years)

Participants reported that they did not use online food delivery services with the intent of purchasing healthy food. Participants told us that they expected takeaway food to be unhealthy and that online food delivery services facilitated access to this food. This perspective influenced the types of food that participants were willing to purchase through online food delivery services. For example, pizza (seen as unhealthy) was appropriate but a salad (seen as healthy) was not. Moreover, participants recognised that if they wanted to consume healthy food, they would most likely cook for themselves.

Participants stated that takeaway food had social, cultural, and behavioural value. For many, purchasing and consuming takeaway food at the end of the working week signified the start of the weekend, which was seen as a time for relaxation and celebration. This tradition was carried forward from childhood, with Friday night referred to as “*takeaway night*”. For participants, using an online food delivery service allowed them to maintain, yet digitalise, traditions.

“*It’s always a weekend thing, besides it being a convenient, really quick way of accessing food that is filling and tastes nice, for me, it marks the end of a work week*.” Participant 4 (Female: 30-39 years)

Participants reported that in some situations consuming takeaway food as a group could be a way to socialise. This was especially the case during life transitions such as leaving home to start university.

“*When you move out you’re concentrating on making friends and getting a takeaway was quite an easy way for everyone to sit down around the table and socialise and to have drinks*.” Participant 14 (Female: 20-29 years)

Participants did not value online food delivery services to the same extent that they did takeaway food. This perspective reinforced that online food delivery services were primarily used to satisfy takeaway food purchasing needs.

“*If Just Eat as an entity disappeared, or all online takeaways disappeared, I wouldn’t be upset* […] *it’s a luxury, it makes life easier*.” Participant 9 (Male: 30-39 years)

#### Less effort for more convenience

Participants reported that it took little effort to use online food delivery services because they receive information about all food outlets that will deliver to them on a single platform. Additionally, participants valued the opportunity to save payment details, previous orders, and favourite food outlets for future use. Participants also informed us that they had a greater number of food outlets and a more diverse range of foods and cuisines to choose from compared with other purchasing formats. Due to the number of accessible food outlets, the selection process was not always fast. Nonetheless, participants indicated that online food delivery services make purchasing takeaway food easier and more convenient than other purchasing formats where information is less readily available.

“Y*ou’ve got all of the different options laid out in front of you, it’s like one resource where everything is there and you can choose and make a decision, rather than having to pull out leaflets from a drawer or Google different takeaways in the area. It’s all there and it’s all uniform and it’s in one place*.” Participant 3 (Female: 20-29 years)

“*I can pick through a whole wide selection rather than being limited to the few takeaways down on my road or having to drive somewhere*.” Participant 21 (Male: 20-29 years)

Participants emphasised that smartphone applications had been optimised to enhance this experience.

“*I guess it’s the convenience of just being able to open the app on my phone, and not have to go searching for menus or phone numbers and checking if places are open. So yeah, it’s the convenience*.” Participant 15 (Male: 40-49 years)

“*For me it’s just the ease of going on, clicking what you want, paying for it and it arriving. You don’t have to move, you don’t have to cook, you don’t have to think, it’s just there ready to go, someone’s doing the hard work for you*.” Participant 1 (Female: 20-29 years)

However, greater convenience was not always advantageous. Some participants were concerned that convenient and easy access to takeaway food through online food delivery services might have negative consequences for health and other things.

“*It’s quite addictive in the way that it’s just so convenient to order. I’m not making stuff fresh at home, and I’m eating unhealthier*.” Participant 21 (Male: 20-29 years)

“*I think it adds to a general kind of laziness that is not good for people really. If you actually got up and went for a walk to go and get this food, at least there’s a slightly positive angle there*.” Participant 17 (Male: 30-39 years)

“*The convenience is not necessarily a positive thing, these apps can be abused because it’s so easy to access foods*.” Participant 10 (Female: 20-29 years)

#### Saving money and reallocating time

Participants were price-sensitive and valued the opportunity to save money. When discussing financial aspects of online food delivery service use, participants referred to special offers they had received by email or through mobile device push notifications. Participants recognised that direct discounts (e.g. 10% off), free items (e.g. free appetizers on orders over £20.00), free delivery (e.g. on orders over £30.00), or time-limited price-promotions (e.g. 40% off all orders for the next three-hours) can justify takeaway food purchasing and online food delivery service use.

“*Getting a takeaway is always a treat, every time I do it I know I shouldn’t but then basically I’m convinced to treat myself, if there’s a discount I’m much more likely to do it because I don’t feel like it’s such a waste of money*.” Participant 18 (Male: 20-29 years)

Participants recognised takeaway food as a distinct food category. Nevertheless, they appreciated that that they could use online food delivery services to purchase ‘restaurant food’. Since this food is usually accompanied by a complete dining experience that online food delivery services cannot replicate, participants expected to spend less on this food purchased online compared to when they dined inside a restaurant.

“*Some restaurants deliver through Deliveroo,* [places] *where you can sit down and have an experience, a dining experience, well that’s different* […] *you might go there for the dining experience*.” Participant 4 (Female: 30-39 years)

“*Sometimes I’m deterred from using Uber Eats because I noticed that the restaurants increase their prices if you buy it through them rather than directly* […] *I don’t want to pay over £10 for a takeaway dish, whereas I would pay that if I ate at a restaurant*.” Participant 3 (Female: 20-29 years)

Although participants considered the price of food when deciding which outlet to order from, they traded money for time. Participants compared the time they would spend cooking or travelling to takeaway food outlets with the time taken to place orders through online food delivery services plus the tasks they could complete whilst waiting for meal delivery. Paying a delivery fee to have the opportunity to use time that would not have otherwise been available was acceptable.

“*Yeah, it costs money but at the same time we’re getting more time with the kids, and more time to do other stuff, so it’s absolutely fine as far as I’m concerned*.” Participant 9 (Male: 30-39 years)

However, some participants were unsure about the appropriateness of paying to have food delivered as it might be unfair to delivery couriers.

“*I don’t feel like it’s necessarily right to make a delivery driver drive two minutes up the road just because I can’t be bothered to go and collect something that’s not very far away*.” Participant 10 (Female: 20-29 years)

#### Online food delivery service normalisation

Participants had positive previous experiences of using online food delivery services. These experiences influenced future custom and contributed to an overall sense that using this purchasing format was now a normal part of living in a digital society. Some participants referred to watching television online to exemplify this point.

The normalisation of using online food delivery services was particularly evident when MK prompted participants to think about the term ‘takeaway food’. Participants often referred to online food delivery services in the first instance and saw them as synonymous with takeaway food.

“*If you were to say ‘takeaway food’ I’d pull out my phone and I’d open one of the apps and say ‘okay, what should we order’, I wouldn’t say ‘oh let’s go to this road’, or ‘let’s go to that road’, I’d say ‘yeah, let’s look on the app’*.” Participant 21 (Male: 20-29 years)

For participants in our study, using online food delivery services replaced purchasing takeaway food in other ways. This perspective was linked to habitual takeaway food purchasing and sociocultural values. Participants rarely purchased takeaway food outside of set routines (for example only doing so at the weekend) because they did not think it was appropriate. As a result, participants reported that they had a limited number of opportunities to use multiple purchasing formats and thus increase their existing levels of consumption.

#### Maintained home food practices

Most participants were responsible for cooking at home, enjoyed doing so, and said they were competent at it. Nonetheless, cooking at home required personal effort and being “*lazy*” or “*tired*” or “*having nothing in the cupboards*” was used as a justification for using online food delivery services.

“*I cook, when I’m not using these apps I cook and prepare food for myself*, *it’s just on the odd occasion I might be feeling tired or want something different* […] *the rest of the time, I’m quite happy to cook*.” Participant 10 (Female: 20-29 years)

Despite the apparent normalisation of using online food delivery services, participants did not feel that they would ever completely eliminate cooking at home. Most participants consumed home cooked food daily, whereas they consumed takeaway food less frequently. This contributed to the view that these two types of food were different. As a result, participants used online food delivery services to purchase food they could not or would not cook at home; for a break from normality, and as a “*cheat*” or “*treat*”.

## Discussion

### Summary of findings

To our knowledge, this is the first published study in the public health literature to investigate experiences of using online food delivery services from the perspective of frequent customers.

Participants recognised that their location and the time of day meant that they could often access different types of food through multiple purchasing formats, at the same time. Participants stated that purchasing takeaway food was appropriate in many situations and typically favoured using online food delivery services. For many participants, using these services was now part of routines in their increasingly digital lives. As such, using online food delivery services appeared to be synonymous with takeaway food purchasing. This meant that participants expected food sold online to be unhealthy and that it was inappropriate to purchase healthy food in this manner. Participants consistently thought about how features of online food delivery services were an advantage or a drawback within the context of their location at any given point in time. This was a complex and dynamic thought process. Participants described how the advantages of these services were a strong enough reason to continue use, overcoming drawbacks such as the acknowledged unhealthfulness of takeaway food. Participants reported that using online food delivery services involved little effort as they were provided with food outlet information, menus, and payment facilities on one platform that had been optimised for use. Moreover, although the cost of food was an important consideration for participants, they were willing to pay a fee in exchange for the opportunity to complete tasks whilst waiting for meal preparation and delivery. Finally, using online food delivery services substituted purchasing takeaway food in other ways. Nevertheless, participants reported that cooking at home was a distinct food practice that occurred more frequently and was irreplaceable.

### Interpretations

Participants described sociocultural values assigned to takeaway food. These values are proposed to develop from previous experiences [38, 39]. For our participants, purchasing takeaway food at the weekend was a traditional routine that celebrated the end of the working week. In the past, this tradition might have meant visiting food outlets in the neighbourhood food environment. However, online food delivery services are now used and favoured. Since participants reported that it was takeaway food in and of itself that was a fundamental reason for seeking out online food delivery services, it is reasonable to conclude that sociocultural values linked to this food exist, and transfer, across purchasing formats.

Food purchasing has been recognised as situational and made in the context of place and time [40, 41], with convenience reported as a consistent consideration [42]. Participants in our study reported that takeaway food was appropriate in many situations and acknowledged that it could often be accessed through multiple purchasing formats. Using one purchasing format over another came after considering multiple factors, including the level of effort required to find a suitable food outlet and place orders. As using online food delivery services took little effort, this purchasing format was often most convenient. However, participants were clear that although their decision had seemingly been made, it could be changed, especially if an online food delivery service feature that was supposedly an advantage became a drawback. For example, if estimated delivery times were too long or delivery fees were too high an alternative option would be considered. Our findings support that the decision about if and how to purchase takeaway food is dynamic and influenced by place and time [32].

Food access has previously been summarised within the domains of availability, accessibility, affordability, accommodation, and acceptability [15]. Although Caspi and colleagues described these domains in the context of physical food access, they are applicable to digital food environments. Broadly speaking, our research investigated the ‘acceptability’ of using online food delivery services, and participants made explicit reference to the domains of food ‘accessibility’ and ‘affordability’.

For example, participants told us that one particularly valuable aspect of using online food delivery services was the ability to access a greater number of food outlets compared with other purchasing formats. This finding speaks to our previous research that found a positive association between having the highest number of food outlets accessible online and any use of online food delivery services in the previous week amongst adults living in the UK [17]. The experiences of using online food delivery services reported in the current study support the possibility that having more food outlet choice contributes to the decision to adopt, and maintain, use of these services rather than necessarily increasing the frequency in which they are used. Other features of online food delivery services, such as having information about each of the accessible food outlets on one platform, likely amplify the perceived benefit of greater food outlet access. Notably, however, access to an increased number of food outlets was not always advantageous. This finding recognises a general awareness about the negative aspects of takeaway food consumption, previously captured from the perspectives of young adults in Australia and Canada [38, 43].

Participants also discussed how the price of food influenced their use of online food delivery services. This reflects that food affordability is a fundamental purchasing consideration [32]. Beyond this, our findings provide insight into actions that food outlets registered to accept orders online might take to attract customers. Given that online food delivery service customers can often select from multiple food outlets at the same time, food outlets might aim to compete with one another by lowering the price of food sold or by introducing price-promotions in an attempt to capitalise on customer demand. Particularly in the case of the latter, participants acknowledged the importance of price-promotions. Previous evidence shows that price-promotions contribute to unhealthy food purchasing practices [44, 45]. Access to price-promotions through online food delivery services has not been systematically documented. However, it is possible that their availability is positively associated with the number of food outlets accessible online. Since both price-promotions and the number of food outlets accessible online appear to influence online food delivery service use, the possibility of interaction between them is concerning for overall consumption of food prepared out-of-home, and subsequently, diet quality and health.

In some cases, participants reported that they used online food delivery services because they did not have time to cook at home. A number of tasks, including household chores, work, travel, and childcare, can limit the time available for, and take priority over, home cooking [46]. Using online food delivery services (and paying associated delivery fees) instead of cooking at home allowed participants in our study to complete non-food related tasks whilst waiting for meal preparation and delivery. Due to sociocultural values and perceived ‘rules’ about how frequently takeaway food 'should' be purchased, participants did not see online food delivery services as a complete replacement for cooking at home. Nevertheless, even partial replacement has implications for diet quality and health, especially since the food available and purchased online was acknowledged as unhealthy by participants in the current study.

### Possible implications for public health and future research

Participants reported that using online food delivery services had mostly substituted, not supplemented, their use of other purchasing formats. Given the perspectives of participants in our study, an increasing number of food outlets could be registering to accept orders online to supply an apparent customer demand. Further research is required to understand the extent to which customer demand is driven by food outlet accessibility, and vice versa.

Participants in our study reported that despite using online food delivery services frequently, their overall takeaway food consumption had remained the same. We do not yet know if this perception would be reflected in objective assessment of takeaway food consumption. Further research that quantifies the use of multiple purchasing formats and takeaway food consumption over time is required to understand the potential public health implications as a result of using online food delivery services. Although evidence from Australia suggests that food sold through online food delivery services tends to be energy-dense and nutrient-poor [47], this has not been established in the UK, to our knowledge. Nor does it necessarily reflect the balance of what food is purchased. Objective assessment of the nutritional quality of foods available, and purchased, through online food delivery services in the UK could be the focus of future research. This evidence will help to better understand the extent to which public health concern is warranted.

With a few exceptions, food sold through online food delivery services is prepared in food outlets that are also physically accessible in the neighbourhood food environment [13]. From a public health perspective, this reinforces the intrinsic link between neighbourhood and digital food environments [48]. Therefore, public health interventions adopted in the neighbourhood food environment may also influence the digital food environment. For example, urban planning policies have been adopted to prevent new takeaway food outlets from opening in neighbourhoods [49]. By extension, this stops new food outlets from becoming accessible online. Other public health interventions that operate synergistically between physical and digital food environments might be increasingly required in the future. It will also be vital for any future interventions to consider how the geographical coverage of online food delivery services expands neighbourhood food outlet access [50], potentially undermining the effectiveness of interventions adopted in the neighbourhood food environment. Doing so would help address concerns that these services increase access to food prepared out-of-home [4, 13]. Interventions of this nature could be particularly important in more deprived areas that have the highest number of accessible food outlets across multiple purchasing formats [16, 51].

Participants recognised that online food delivery services provide access to takeaway food that was associated with being unhealthy. Participants were aware that they *could* purchase healthy food through online food delivery services, but this did not mean that they *would*. From a public health perspective, this finding indicates that the success of interventions intended to promote healthier takeaway food purchasing through online food delivery services might be limited by existing sociocultural values if they are not taken into consideration. A possible way to navigate this would be to improve the nutritional quality of food available online without necessarily making any changes salient. Interventions of this nature include healthier frying practices and reduced food packaging size [52, 53]. Although these interventions were acceptable and feasible when implemented inside takeaway food outlets [54], further investigation is required to understand the extent to which they are appropriate in the context of online food delivery services. Changing the types of food available to purchase through online food delivery services could also lead to improved food access for those with limited kitchen facilities at home or limited mobility.

Public health interventions intended specifically for online food delivery services could also be developed. Potential approaches include preferential placement of healthy menu items, introducing calorie labelling and offering healthier food swaps. Embedding these approaches within existing online food delivery service infrastructures would allow implementation to be uniform [55], and their implementation could be optimised to enhance customer awareness and interaction. The potential success of approaches of this nature requires exploration. Nevertheless, in February 2022, the UK Behavioural Insights Team (formerly of the UK Government) published a protocol to investigate approaches to promoting the purchase of lower energy density foods through a simulated online food delivery service platform [56].

Price-promotions influenced and justified the use of online food delivery services. Legislation to restrict the use of volume-based price-promotions (e.g. buy-one-get-one-free, 50% extra free) on less healthy pre-packaged food sold both in-store and online were due to be introduced in England in October 2022 [57]. However, the introduction of this legislation has now been delayed. Although hot food served ready-to-consume was due to be excluded, given what is known about the impact of price-promotions on purchasing other food [58], and our participants’ description of the importance of price-promotions on their purchasing practices, extension of these restrictions to hot food served ready-to-consume might be warranted. Understanding how price-promotions influence food purchased from online food delivery services represents a first step to understand the need for future regulation.

### Limitations

We recruited participants through two social media platforms, which means that our study sample was formed from a subset of all social media users. However, online recruitment was appropriate since we wanted to understand experiences of using a digital purchasing format. Moreover, the participants we recruited were mostly highly educated, potentially reflecting reported online food delivery service use amongst this socioeconomic group [22, 23]. After 12 telephone interviews we acknowledged this and adjusted our recruitment strategy to ensure a more balanced sample with respect to level of education. Nevertheless, future research should explore the perspectives of frequent online food delivery service customers with lower levels of education, since it is possible that they have different reasons for using these services. Although we did not recruit infrequent online food delivery service customers or non-customers, they would not have been well-positioned to help us investigate our study aims. However, since we have described experiences of using online food delivery services from the perspective of frequent customers, future work should seek to understand perspectives of non-customers, customers who use them less frequently, and customers who use them for specific reasons.

As the first study in the public health literature to investigate frequent customer experiences of using online food delivery services, we chose a descriptive methodological orientation. Our descriptive approach meant that we did not investigate the underlying meaning of the language used by participants, however, this was not aligned with our aims. Furthermore, our descriptive methodological orientation allowed us to use codebook thematic analysis and include multiple researchers in analysis. Coding a 10% sample of interviews transcripts and discussing analytic themes would have been less appropriate with reflexive approaches to thematic analysis [34, 35, 59], but assisted with our interpretations.

We conducted fieldwork during the early stages of the COVID-19 pandemic, which might have altered the recent experiences of online food delivery service use and participant perspectives. However, MK asked participants to think about the time before the COVID-19 pandemic and reflected on their ability to do so. This reflexivity is in line with established practices regarding qualitative rigour [20, 60], and allowed us to determine when it would be most appropriate to stop fieldwork. Nonetheless, we acknowledge the possibility that food-related practices have changed during the COVID-19 pandemic. As a result, it is possible that online food delivery services are now used for different reasons, both initially and over time, and by individuals with different sociodemographic characteristics than those in our study.

## Conclusions

We used telephone interviews with frequent online food delivery service customers to investigate experiences of using this purchasing format. We found that the context of place and time influenced if and how takeaway food would be purchased. Online food delivery services were often seen as most appropriate. In part, this was due to the opportunity to access advantages not available through other purchasing formats, such as efficient and convenient ordering processes that had been optimised for customers. Fundamentally, however, online food delivery services provide access to takeaway food, which despite being acknowledged as unhealthy, has strong sociocultural value. There was a consistent awareness that some advantages of online food delivery services may also be drawbacks. Despite this, the drawbacks were not sufficiently negative to stop current or future online food delivery service use. Finally, price-promotions justified online food delivery service use and made this practice appealing. Public health interventions that seek to promote healthier food purchasing through online food delivery services may be increasingly warranted in the future. Approaches might include increasing the healthiness of the food available whilst maintaining sociocultural values and expectations, and extending restrictions on price-promotions to hot food prepared out-of-home.

## Supplementary Information

**Additional file 1: Box A1.** Names of Subreddits used during participant recruitment. **Box A2.** Final telephone interview topic guide.

## Data Availability

Processed and anonymised qualitative data from this study is available from the corresponding author upon reasonable request. Additional raw data related to this publication cannot be openly released; the raw data contains interview audio containing identifiable information.
