# Supplementary material for: Investigating experiences of frequent online food delivery service use: a qualitative study in UK adults
Source: BMC Public Health. 2022 Jul 16;22:1365. doi: 10.1186/s12889-022-13721-9 (PMC9287535; doi:10.1186/s12889-022-13721-9)
Supplement: Supplementary file 1 — Additional file 1: Box A1. Names of Subreddits used during participant recruitment. Box A2. Final telephone interview topic guide. [file 12889_2022_13721_MOESM1_ESM.docx]

| Box A1: Names of Subreddits used to post recruitment materials | |
| --- | --- |
| **UK cities** |  |
| Birmingham |  |
| Bradford |  |
| Brighton |  |
| Bristol |  |
| Cornwall |  |
| Coventry |  |
| Edinburgh |  |
| Glasgow |  |
| Leeds |  |
| Leicester |  |
| Liverpool |  |
| London |  |
| Manchester |  |
| Newcastle-upon-Tyne |  |
| Sheffield |  |
| **UK or food based** |  |
| AskUK |  |
| GhostKitchens |  |
| JustEatUK |  |
| Unitedkingdom |  |

| Box A2: Final telephone interview topic guide |
| --- |
| **PRE-AMBLE AND INTRODUCTION** |
| - Participant age, gender, marital status and occupation confirmed. |
| - Definitions for ‘online food delivery services’ (online takeaways) and ‘food prepared out-of-home’ (takeaway food) discussed and confirmed. |
| **PATTERNS OF TAKEAWAY FOOD PURCHASING** |
| - What are the different ways that you have purchased takeaway food in the past 12 months? |
| - What do you think about when you are going to buy takeaway food? Maybe you could touch on things like how you choose the way that you are going to buy it, and the types of things you think about. |
| - Thinking about before March, when and why would you typically buy takeaway food? |
| - - What is it about these days or times? |
| **ONLINE FOOD DELIVERY SERVICE ADOPTION AND USE** |
| (*If not discussed during conversation so far*)   - Which online takeaways have you used before? |
| - When did you start using ‘them’? [Note: ‘them’ refers to the name of the online takeaway] |
| - What made you first start using them? |
| - When might you use them instead of another purchasing format? |
| - How do you choose which food outlet you are going to buy from when you use them? |
| - Do you think using an online takeaway changes the food outlets you buy from? If so, how? |
| **PERCEIVED BENEFITS AND DRAWBACKS OF ONLINE TAKEAWAYS** |
| - What do you think are the most important features of online takeaways? What are you expecting when you use them? |
| - How do you think these features might be different compared to other ways of buying takeaway food? |
| - Still thinking about before March, in your opinion, what are the main benefits of using online takeaways? |
| - What is not so good about using online takeaways? |
| - - You mentioned some things that might not be so good about using online takeaways, so why do you keep using them – what is it about online takeaways? |
| **BROADER BEHAVIOURS AND PERCEPTIONS** |
| - What do you think about when you hear the term ‘takeaway food’? |
| - What do you think about the types of food you can buy through online takeaways? |
| - Is the food you can buy online any different from the food you can buy in other ways? |
| - Thinking about all of the different places that you can buy takeaway food from near where you live, how does that compare to what you can get through online takeaways? |
| - How do you think using an online takeaway has changed other ways of buying takeaway food? - How do you think you will you buy takeaway food in the future? |
| - How does using online takeaways fit in with other things like cooking food at home? |
| **SUMMARY** |
| - Important points identified throughout interview reframed, and accuracy confirmed. |
| - Participant asked to discuss any points in more detail or if anything had not been asked but should have been. |
